# Moderate agreement between self-reported stroke and hospital-recorded stroke in two cohorts of Australian women: a validation study

**DOI:** 10.1186/1471-2288-15-7

**Published:** 2015-01-23

**Authors:** Caroline A Jackson, Gita D Mishra, Leigh Tooth, Julie Byles, Annette Dobson

**Affiliations:** Centre for Longitudinal and Life Course Research, School of Public Health, University of Queensland, Herston, QLD 4006 Australia; Research Centre for Gender, Health and Ageing, University of Newcastle, Callaghan, NSW 2308 Australia

**Keywords:** Epidemiology, Stroke, Cerebrovascular disease, Validation studies, Self-report, Hospitalisation

## Abstract

**Background:**

Conflicting findings on the validity of self-reported stroke from existing studies creates uncertainty about the appropriateness of using self-reported stroke in epidemiological research. We aimed to compare self-reported stroke against hospital-recorded stroke, and investigate reasons for disagreement.

**Methods:**

We included participants from the Australian Longitudinal Study on Women’s Health born in 1921–26 (n = 1556) and 1946–51 (n = 2119), who were living in New South Wales and who returned all survey questionnaires over a defined period of time. We determined agreement between self-reported and hospitalised stroke by calculating sensitivity, specificity and kappa statistics. We investigated whether characteristics including age, education, area of residence, country of birth, language spoken at home, recent mental health at survey completion and proxy completion of questionnaire were associated with disagreement, using logistic regression analysis to obtain odds ratios (ORs) with 95% confidence intervals (CIs).

**Results:**

Agreement between self-report and hospital-recorded stroke was fair in older women (kappa 0.35, 95% CI 0.25 to 0.46) and moderate in mid-aged women (0.56, 95% CI 0.37 to 0.75). There was a high proportion with unverified self-reported stroke, partly due to: reporting of transient ischaemic attacks; strokes occurring outside the period of interest; and possible reporting of stroke-like conditions. In the older cohort, a large proportion with unverified stroke had hospital records of other cerebrovascular disease. In both cohorts, higher education was associated with agreement, whereas recent poor mental health was associated with disagreement.

**Conclusion:**

Among women who returned survey questionnaires within the period of interest, validity of self-reported stroke was fair to moderate, but is probably underestimated. Agreement between self-report and hospital-recorded stroke was associated with individual characteristics. Where clinically verified stroke data are unavailable, self-report may be a reasonable alternative method of stroke ascertainment for some epidemiological studies.

**Electronic supplementary material:**

The online version of this article (doi:10.1186/1471-2288-15-7) contains supplementary material, which is available to authorized users.

## Background

Epidemiological studies often rely on self-report questionnaires to ascertain disease occurrence. This is a valuable method of ascertainment, especially in the absence of disease-specific population registers, since it is cost-efficient and feasible in large study populations. Much of our current knowledge on the incidence and aetiology of stroke generally derives from studies in clinical settings where strokes are carefully diagnosed and phenotyped. The collection of detailed clinical data allows thorough investigation of particular risk factors, stroke subtypes and outcome after stroke. However, whilst these studies are rich in clinical data, they collect far less information on other important aspects, including socioeconomic, lifestyle, psychosocial and environment/social context factors. In addition, these data are rarely collected prospectively in clinical studies, prior to stroke occurrence. Existing population-based studies that have the advantage of prospective data collection (and in the case of longitudinal studies, repeated data collection) may be used to study the contribution of these non-clinical factors to stroke aetiology and outcome. Similarly, such studies may be beneficial in studying trends in prevalence and incidence. However these studies may not always have access to clinically verified incident stroke, relying instead on self-report questionnaires. It is therefore crucial to establish the validity of self-reported stroke, especially given the complexity of diagnosing this disease.

Self-report of conditions that are well defined and/or easier to diagnose, such as cancer and diabetes, generally have a high positive predictive value (PPV) [1–3]. However, agreement is usually lower for diseases such as stroke that are more complex in their diagnosis. Stroke is a heterogeneous disease with symptoms ranging from mild to severe, and there is no definitive diagnostic test. Some or all of the symptoms may resolve prior to medical consultation, which can further complicate the diagnosis. Stroke largely affects older people and can impact on cognitive function, both of which may cause reduced recall capacity and accuracy of self-reporting. Furthermore, transient ischaemic attacks (TIA), where symptoms last less than 24 hours, are often misunderstood to be, and thus reported as, strokes. This may be partly due to TIAs being frequently referred to as ‘mini strokes’. Nevertheless, in some settings it is not possible to ascertain disease occurrence using alternative sources of information, such as health records or hospital discharge data. In addition, universal access to *all* health records of an individual is often impossible. For example, in Australia, linked admitted hospital patient data are not yet available nationally (only for some states), outpatient data are not included in routine hospital data, and there is no routine linkage to primary care records.

Reports of the validity of self-reported stroke vary considerably, from low/moderate [4–6], to good/very good [3, 7–9]. Some of this variation is most likely due to differences in settings, age groups, gender and the ‘gold standard’ against which self-reported stroke is verified. Conflicting recommendations arising from these studies [4, 5, 7, 9] have created uncertainty about the appropriateness of using self-reported stroke in epidemiological research. Whilst some authors suggest that self-reported stroke is a valid method of assessing stroke prevalence, others recommend that self-reported stroke should be used with considerable caution, or should only be used in combination with other ascertainment methods.

However, existing studies have generally compared self-reported stroke to other ascertainment methods without identifying the potential reasons for any observed discrepancy. A better understanding of the reasons for discrepancies will further inform the appropriateness and implications of using self-reported stroke data. In this study, we determined agreement between self-reported and hospital-recorded stroke in two age-groups of women. We identified individual-level factors influencing agreement and investigated reasons for disagreement.

## Methods

### Study setting

We included participants from the Australian Longitudinal Study on Women’s Health (ALSWH), a national population-based study of women born in 1921–26, 1946–51 and 1973–78. Women were randomly selected from the Medicare database, which covers all citizens and permanent residents of Australia, including refugees and immigrants, with intentional oversampling of women living in rural and remote areas. Women were surveyed in 1996, followed up in 1998 (1946–51 cohort) and 1999 (1921–26 cohort), and subsequently followed up every three years. At baseline, the 1921–26 and 1946–51 cohorts included 12,432 and 13,715 women, respectively. Full details of the recruitment and response rates are reported elsewhere [10]. The study participants are linked to the national death register. National linkage of the ALSWH cohorts to other routinely collected data, including hospital-admitted patient data, is underway, with New South Wales (NSW) being the first state in which ALSWH data are linked.

### Study population

We included 3675 women in our analyses. This included 1556 women from the 1921–26 cohort who were alive between survey 3 (2002) and survey 5 (2008) and 2119 women from the 1946–51 cohort who were alive between survey 3 (2001) and survey 6 (2010). Women had to have resided in NSW and returned all survey questionnaires during this time. The study was approved by the Human Research Ethics Committee of the University of Newcastle, the Medical Research Ethics Committee of the University of Queensland and the Departmental Ethics Committee of the Australian Government Department of Health and Ageing.

### Questionnaire data

The surveys collect data on demographic characteristics, health conditions and behaviours. A free-text section allows women to provide additional comments.

Self-reported stroke was defined as having occurred during the period of interest if the participant responded ‘yes’ to the question: “In the past three years have you been diagnosed with or treated for stroke?”

History of hypertension, diabetes and heart disease were self-reported and poor mental health in the four weeks prior to survey completion was determined using the SF36 mental health subscale, with a score of ≤ 52 indicating poor mental health [11].

### Hospital-recorded stroke

Hospital data was available for 2000–2010 and included admission and discharge dates, principal and secondary diagnoses and procedure codes. Strokes were identified by the following International Statistical Classification of Diseases and Related Health Problems 10th revision (ICD-10) codes in principal or secondary diagnosis fields: I60-I60.9, I61.0-I61.9, I63.0-I63.9 and I64. Although women were asked about the occurrence of stroke within the last three years, we anticipated recall error in *when* an event occurred (particularly in the older cohort). Restriction of the hospital admission period to these three-year intervals would have led to over or under-estimated agreement due to recall error in when the stroke occurred and not whether it occurred at all. Stroke was therefore defined as having occurred if the admission date was between the dates of return of survey 3 and survey 6 for the 1946–51 cohort or between return of survey 3 and 5 for the 1921–26 cohort. ICD-10 procedure codes were examined to identify occurrence of brain imaging.

### Statistical analyses

Analyses were performed using Stata version 11.0.

We compared characteristics of women who were eligible for inclusion versus those who were ineligible using Pearson’s chi-squared test or Student’s t-test for continuous variables.

#### Agreement between self-reported and hospital-recorded stroke

We calculated sensitivity, specificity, PPVs and negative predictive values (NPV). We calculated Cohen’s kappa with 95% confidence intervals (CI), using the following interpretation categories: poor kappa agreement, <0.2; fair, 0.21 – 0.40; moderate, 0.41-0.60; good, 0.61-0.80; and very good, 0.81-1.00 [12]. The magnitude of kappa is affected by imbalance between the positive and negative classifications, which occurs when the disease of interest has a low prevalence. To aid interpretation of kappa, we calculated the prevalence index, (a-d)/N, where *a* and *d* are concordant ‘ratings’ and N = total population [13]. A greater prevalence effect leads to a higher prevalence index, greater chance agreement, and consequently, a reduced kappa [13].

#### Association between characteristics and disagreement

We examined associations between individual characteristics and disagreement by calculating unadjusted and adjusted odds ratios using logistic regression. We defined disagreement as false negative or false positive (as summarised in Table 1). In the 1946–51 cohort we examined age, education, area of residence, country of birth, language spoken at home, and recent mental health at survey completion. Due to the small number of women in the ‘disagreement’ group we only included variables that were statistically significant in the univariate analysis in the adjusted model, controlling for age. In the older cohort we also included proxy completion of questionnaire, with the adjusted model including age, area of residence and variables that were significant in the univariate analysis.

**Table 1:** **Agreement between self-reported and hospitalised stroke**

	1921-26 cohort					1946-52 cohort				
			Hospital-recorded stroke					Hospital-recorded stroke		
			Yes	No	Total			Yes	No	Total
	Self-reported stroke	Yes	25	77	102	Self-reported stroke	Yes	11	14	25
		No	7	1447	1454		No	3	2091	2094
		Total	32	1524	1556		Total	14	2105	2119
**Agreement measures**										
Prevalence (self-report)		6.6%					1.2%			
Prevalence (hospital-recorded)		2%					0.7%			
Sensitivity		78.1%					78.6%			
Specificity		94.9%					99.3%			
Positive predictive value		24.5%					44.0%			
Negative predictive value		99.5%					99.9%			
Kappa (95% CI)		0.35 (0.25 to 0.46)					0.56 (0.37 to 0.75)			
Prevalence index		0.91					0.98			

#### Investigation of disagreement

Among women with unverified self-reported stroke, we examined discharge codes of any hospital admissions to identify: transient ischaemic attacks (TIA); diagnoses of possible strokes (or possible ‘stroke mimics’ [14]); sequelae of cerebrovascular disease; and other cerebrovascular disease diagnoses (e.g. occlusion or stenosis of (pre)cerebral arteries). We examined: procedure diagnosis fields, especially for those women in whom a possible ‘stroke mimic’ may have occurred; residence postcodes to identify women living closer to a hospital outside NSW; and questionnaire comments for additional information concerning stroke occurrence.

Finally, we calculated the agreement statistics based on different definitions of self-report and hospital-recorded cerebrovascular disease.

## Results

### Characteristics of study population

**Figure 1:**
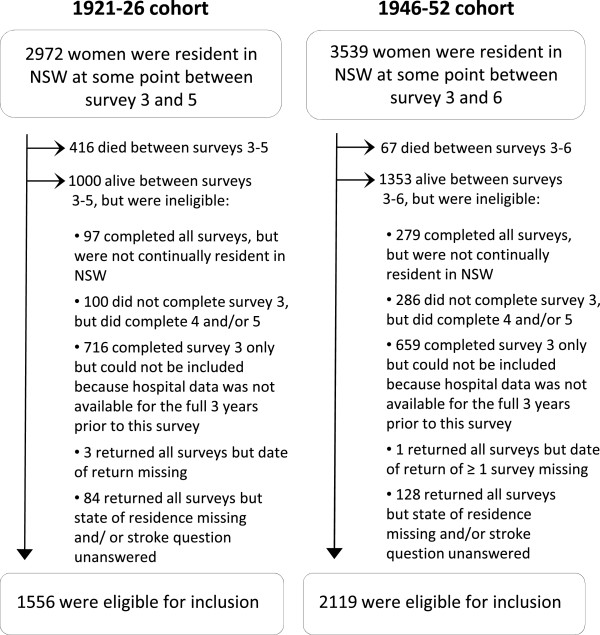
**Flow diagram of included and excluded participants.**

Figure 1 details the reasons why some women who were resident in NSW at some time during the periods of interest were ineligible for inclusion. Eligible women had generally healthier lifestyle behaviours, reported better general health, had a higher education level, and were more likely to speak English at home and to have been born in Australia or an English speaking country, than ineligible women (Additional file 1: Table S1 and Additional file 2: Table S2).

The mean age of women included from the older and mid-age cohorts was 78.2 (±1.5) and 52.5 (±1.5) respectively. Other characteristics are presented in Additional file 1: Table S1 and Additional file 2: Table S2.

### Agreement in the 1921–26 cohort

**Figure 2:**
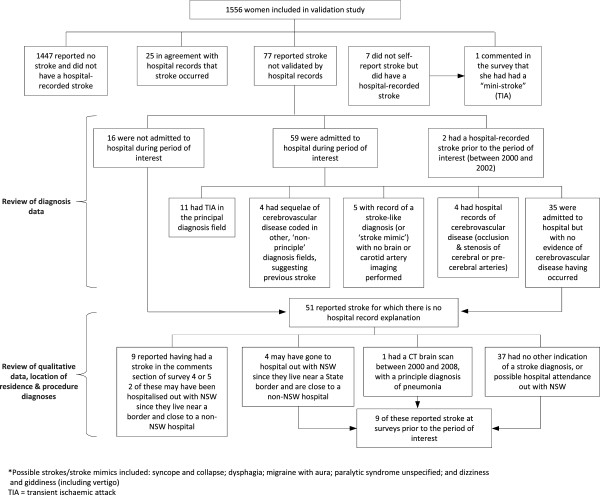
**Flow diagram of investigation of disagreement in the 1921–26 cohort.**

In the 1921–26 cohort, 102 of 1556 women (6.6%) reported stroke. Agreement between self-reported and hospital-recorded stroke was fair (kappa 0.35, 95% CI 0.25 to 0.46; Table 1). The high prevalence index indicates that kappa has been negatively affected and may be underestimated. Specificity and sensitivity were high. The NPV was also high, reflecting the low false negative rate. The PPV was low (24.5%), reflecting a high false positive rate (77 of 102 self-reported strokes were unverified by hospital data). Figure 2 summarises our investigation of women for whom there was disagreement. Of the 77 women with unverified self-reported stroke, 77% were admitted to hospital. The key findings were: two hospital-recorded strokes occurred outside the period of interest; 11 women (14%) had a TIA; four had admissions for sequelae of cerebrovascular disease; five had stroke-like diagnoses; and four had diagnoses of occlusion or stenosis of cerebral or pre-cerebral arteries. Therefore, about one third of women with unverified self-reported stroke had evidence of cerebrovascular disease from hospital records. Among women with no hospital admission or non-cerebrovascular diagnoses, nine (18%) provided additional comments on the stroke occurrence in the questionnaire.

### Agreement in the 1946–51 cohort

**Figure 3:**
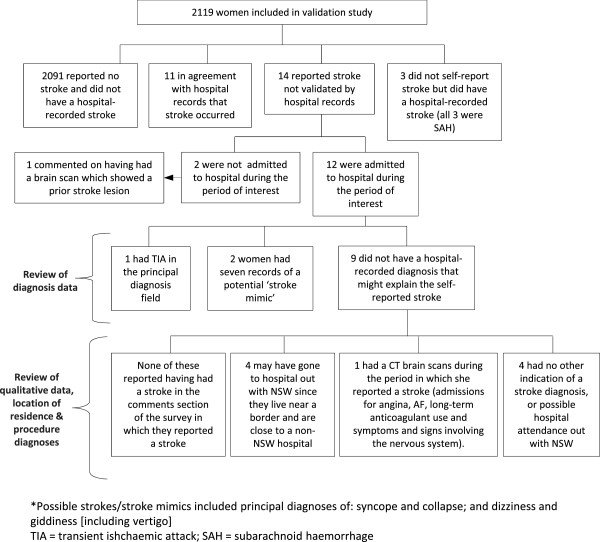
**Flow diagram of investigation of disagreement in the 1946–52 cohort.**

In the 1946–51 cohort, 23 of 2119 women (1.1%) reported a stroke. Agreement was higher than in the older cohort (kappa 0.56, 95% CI 0.37 to 0.75; Table 1). Again, the high prevalence index indicates that kappa has been negatively affected. The NPV was high, reflecting the low false negative rate. The PPV was relatively low (44.0%), albeit higher than in the older cohort, with 14 of 23 self-reported strokes not verified by hospital data. Figure 3 summarises the results of the investigation of women for whom there was disagreement. Most were admitted to hospital during the period of interest, with one (7%) having a TIA, and two a stroke-like diagnosis. One woman had multiple records of admission for long-term anticoagulant use and was admitted for neurological symptoms and signs on one occasion. One woman who reported a stroke (but had no hospital admission records) commented on having had a brain scan that had apparently ‘shown that she had had a mini-stroke at some point’.

### Characteristics associated with disagreement

In the older cohort, a tertiary or trade educational qualification was associated with decreased odds of disagreement compared with having high school education (OR 0.24, 95% CI 0.09 to 0.67). In the 1946–52 cohort, having no formal qualifications was associated with increased odds of disagreement (OR 4.21, 95% CI 1.33 to 13.32). Recent poor mental health was associated with disagreement in both cohorts (1921–26 cohort: OR 2.41, 95% CI 1.20 to 4.81); 1946–52 cohort: OR 3.20, 95% CI 1.08 to 9.46; Table 2). In the older cohort, proxy completion of the questionnaire was associated with increased disagreement in unadjusted, analyses (OR 3.82, 95% CI 1.27 to 11.5), but became non-significant in adjusted analyses (OR 2.36, 95% CI 0.65 to 8.58).

**Table 2:** **Characteristics associated with disagreement in self-report compared with hospital-recorded stroke**

	1921-26 cohort				1946-51 cohort			
			OR (95% CI)				OR (95% CI)	
Characteristic	Agreement (N = 1472) n (%)	Disagreement (N = 84) n (%)	Unadjusted	Adjusted	Agreement (N = 2102) n (%)	Disagreement (N = 17) n (%)	Unadjusted	Adjusted
Age (mean ± SE)	78.1 (±1.4)	78.4 (±1.56)	1.11 (0.96 to 1.30)	1.13 (0.97 to 1.32)	52.5 (±1.4)	52.2 (±1.7)	0.87 (0.62 to 1.22)	0.87 (0.62 to 1.23)
Education								
High school education	814 (56.3)	52 (65.0)	1.00	1.00	1021 (48.8)	6 (35.3)	1.00	1.00
No formal education/primary only*	365 (25.2)	24 (30.0)	1.03 (0.62 to 1.70)	0.97 (0.58 to 1.63)	217 (10.4)	6 (35.3)	**4.71 (1.50 to 14.7)**	**4.21 (1.33 to 13.32)** ^**§**^
Tertiary/trade qualification	268 (18.5)	4 (5.0)	**0.23 (0.08 to 0.65)**	**0.24 (0.09 to 0.67)** ^**‡**^	854 (40.8)	5 (29.4)	1.00 (0.30 to 3.28)	0.80 (0.26 to 2.86)
Area of residence								
Urban	658 (44.7)	34 (40.5)	1.00	1.00	795 (38.0)	6 (35.3)	1.00	-
Rural or remote	814 (55.3)	50 (59.5)	1.19 (0.76 to 1.86)	1.08 (0.68 to 1.72)	1295 (62.0)	11 (64.7)	1.13 (0.41 to 3.06)	-
Country of birth								
Australia/other English	1377 (93.5)	77 (91.7)	1.00	-	1956 (93.1)	16 (94.1)	1.00	-
background								
Other	95 (6.5)	7 (8.3)	0.76 (0.34 to 1.69)	-	146 (6.9)	1 (5.9)	0.84 (0.11 to 6.36)	-
Language spoken at home								
English	1422 (96.7)	80 (95.2)	1.00	-	1991 (96.2)	16 (100)	-	-
Other	50 (3.4)	4 (4.8)	1.42 (0.50 to 4.04)	-	79 (3.8)	0 (0)	-	-
Recent poor mental^†^								
No	1354 (94.2)	70 (85.3)	1.00	1.00				
Yes	83 (5.8)	12 (15.0)	**2.80 (1.46 to 5.36)**	**2.41 (1.20 to 4.81)** ^**‡**^	222 (10.6)	5/16 (31.3)	**3.85 (1.33 to 11.18)**	**3.20 (1.08 to 9.46)** ^**§**^
Proxy completion of survey								
No	1453 (98.7)	80 (95.2)	1.00	1.00	-	-	-	-
Yes	19 (1.3)	4 (4.7)	**3.82 (1.27 to 11.5)**	2.36 (0.65 to 8.58)	-	-	-	-

*In the 1946-51cohort, this category reflects women with no formal qualifications.

^†^Where stroke was self-reported, the mental health score from that same survey was used; for hospital-recorded strokes where stroke was not self-reported, the mental health score from the survey subsequent to the admission was used; where there was neither hospital-recorded nor self-reported stroke, the first available mental health score from the included surveys was used.

^‡^p ≤ 0.01.

^§^p < 0.05.

CI = confidence interval; OR = odds ratio; SE = standard error.

NB: bold text indicate statistically significant odds ratios.

### Scenarios of agreement

When we allow for the misreporting of TIAs as strokes, and admission for chronic stroke, agreement between self-reported and hospital-recorded stroke improves, particularly in the older cohort, with the kappa increasing markedly from 0.35 to 0.54 (Table 3). Agreement in the older cohort improves further when we compare self-reported stroke to hospital-recorded cerebrovascular disease in general and further still when we take into account comments by women which may support a valid stroke occurrence. As already mentioned, kappa values were negatively affected by the low stroke prevalence in this study population and should be interpreted with caution.

**Table 3:** **Scenarios of agreement in the validation of self-reported stroke**

Scenario	Prevalence (self-report)	Prevalence (hospital-recorded)	Sensitivity	Positive predictive value	Kappa (95% CI)	Prevalence index
1921-26 cohort						
A*	6.6%	2.1%	78.1%	24.5%	0.35 (0.25 to 0.46)	0.91
**B** ^**†**^	6.6%	3.1%	86.0%	41.2%	0.54 (0.44 to 0.63)	0.90
**C** ^**‡**^	6.6%	3.4%	86.8%	45.1%	0.57 (0.48 to 0.67)	0.90
**D**	6.6%	4.0%	88.7%	53.9%	0.65 (0.57 to 0.74)	0.89
1946-52 cohort						
A*	1.2%	0.7%	78.6%	44.0%	0.56 (0.37 to 0.75)	0.98
**B** ^**†**^	1.2%	0.7%	80.0%	48.0%	0.60 (0.42 to 0.78)	0.98

*Scenario A: Agreement between self-reported and hospital-recorded stroke.

^†^Scenario B: Hospital comparison group includes TIA and sequelae of cerebrovascular disease diagnoses, and hospital–recorded strokes occurring before the period of interest.

^‡^Scenario C: Compares self-reported stroke with any hospital-recorded cerebrovascular disease.

^§^Scenario D: Scenario C, plus includes women as having had a possible stroke if they provided comments on the stroke event in the questionnaire.

## Discussion

In our study, agreement between self-reported stroke and hospital-recorded stroke initially appears fair to moderate. Few women failed to report a stroke that was recorded in hospital records, but a substantial number reported a stroke which was unverified by hospital records. Almost a fifth of women in the older cohort who reported a stroke provided additional comments on the stroke occurrence (sometimes with detailed reference to symptoms and doctor consultations), suggesting that some women may have been diagnosed in a non-hospital setting. Furthermore, women who self-reported unverified strokes often misreported TIAs as strokes, had evidence of chronic stroke, or had evidence of other cerebrovascular disease from discharge diagnoses, suggesting that self-reported stroke may be a reasonable indicator of cerebrovascular disease in general. Our investigation of the individual factors associated with level of agreement suggests that validity may vary with education level and mental health status at time of self-report of stroke.

### Comparisons with previous studies

Our findings are consistent with results of previous studies which generally found low or moderate levels of agreement, with low PPVs (22% to 55%) [4–6, 15]. In contrast, some studies found higher levels of agreement, with higher PPVs [2, 3, 7–9]. However, the ‘gold-standard’ against which self-reported stroke has been compared varies markedly between studies, ranging from hospital discharge or medical record review [2, 4–6, 8] to general practitioner or health centre record review [2, 16, 17] or a range of information including interview and/or clinical assessment of participants [3, 7, 9]; this explains some of the variation and highlights the potential limitations of relying on hospital data only to identify strokes. Interestingly, studies in which multiple sources of information (and not only hospital records) were used to verify self-report generally reported better validity of self-reported stroke than studies that include medical records only for example [3, 7, 9].

Few studies have investigated the influence of participant characteristics on the validity of self-reported stroke. In our study, higher educational level was associated with increased agreement amongst the older cohort, which is consistent with the findings of Okura *et al*. [8], but not with a Norwegian study, which found no such association [7]. We also found that recent poor mental health at time of survey completion was significantly associated with increased odds of disagreement. Engstad *et al*. similarly found that being happy or optimistic during the last two weeks was associated with higher agreement, but this was not statistically significant after adjusting for confounding [7].

Although we did not formally test it, disagreement was higher among the older cohort than the younger cohort in our study. Ageing was also associated with decreased agreement in two other studies [8, 17].

The prevalence of self-reported stroke in our cohort is in keeping with estimates of stroke prevalence in women of these ages from population-based studies in similar high-income countries during the 1990s [16, 18]. The prevalence of stroke as validated by hospital record is considerably lower, suggesting we may underestimate stroke prevalence if we rely solely on this ascertainment method. A recent Australian study found that verification of self-reported cardiovascular disease events (including stroke) by linkage to a state-wide hospital morbidity database gave similar estimates of validity to verification by adjudication of medical records [19]. Although quality of hospital discharge data might vary across Australia, it is reassuring that these routinely available data are accurate in terms of reflecting medical record data and suggests that our findings may not have been significantly affected by errors in discharge data.

### Strengths

Our study has a number of strengths. First, we were able to assess and compare the validity of self-reported stroke in two age groups of women. Our examination of the validity of self-reported stroke in a middle-aged cohort is novel, since previous studies have generally only included an older study population. Second, our study population was large, resulting in a reasonably large number of stroke outcomes among the older cohort. Third, our analyses sought to identify reasons for disagreement between self-reported and hospital-recorded stroke, which extends the existing literature, with many of the existing studies on this topic only assessing agreement without further investigation. Fourth, the ALSWH surveys collect a wealth of demographic and health and well-being data, allowing us to investigate the characteristics associated with validity of stroke reporting, which few studies have explored.

### Limitations

Our study has some limitations. We included women only, and so the results may not be generalizable to men. However, agreement between self-reported and hospital-recorded stroke in our study is in keeping with that observed in a very similar population-based study of older Australian men [15]. Studies reporting agreement by gender found some differences, with the PPV slightly lower in women [5, 6, 16], largely due to lower stroke prevalence. One study reported no association between gender and disagreement [8], whilst another found poorer agreement in women [7].

The limitations of the hospital data, in terms of the period for which data was available imposed some restrictions on our inclusion criteria, leading to the exclusion of women from our analyses. There were some differences between the included and excluded women, especially in the older cohort. Importantly, excluded women had a lower education level than included women. Our finding that a higher education level is associated with greater agreement suggests that excluded women may have been less reliable self-reporters, and thus we may have overestimated general population agreement.

Conversely, we probably underestimated the true validity of self-reported stroke. Firstly, we were unable to verify non-hospitalised strokes. In an Australian community-based study of stroke incidence, 86% of strokes were hospitalised [20]. Given that a certain proportion of the remaining 14% had a non-hospitalised fatal stroke, and assuming a similar hospitalisation rate in NSW, these data suggest that a relatively small proportion (<10%) of women may have had a non-hospitalised stroke. We also could not verify stroke occurrence leading to hospitalisation in another Australian state. This is a particular problem for women who lived close to state borders. However we found that, based on residence postcode, this is likely to affect few participants. Finally, despite being asked about stroke events occurring in the past three years, some women may have reported strokes occurring before 2000, which we could not validate. However, we were able to account for some of these sources of error, by determining ‘best-case’ and ‘worst-case’ estimates of the validity of self-reported stroke.

## Conclusions

Ideally, multiple methods of ascertainment should be used to identify stroke occurrence, but in questionnaire-based research as well as clinical practice, this is often not feasible, resulting in reliance on self-report or routinely available data such as hospital discharge information. Although the latter approach may not identify all strokes, the proportion of false positives will be low, and the direction of any bias more easily determined. This makes it a more attractive, and where the data are available, preferable method of stroke ascertainment compared to self-report. However, the limitations of this method (for example, selection bias) need to be acknowledged and the implications for interpretation considered. Where hospital data are unavailable, self-reported stroke may be a reasonable alternative method of ascertainment for some epidemiological studies, particularly where there is the opportunity to investigate the role of under-researched non-clinical factors that may play an important role in aetiology and outcome. Reassuringly, a recent study demonstrated similarities in stroke incidence and cardiovascular risk factor associations between a study using self-reported stroke and studies that included clinically-verified strokes [21]. Also, recent findings from a population-based study of older men found that rates of recurrent stroke were similar regardless of whether stroke was ascertained by self-report or hospital diagnosis [15]. However, researchers should be aware of the caveats to using self-reported stroke data, especially among studies of older people. Self-reported stroke will inevitably include the reporting of other stroke-like or stroke-related conditions (as well as some non-strokes) and findings should therefore be appropriately interpreted. If research questions relate to cerebrovascular disease in general, then using self-report to ascertain stroke may be less biased.

## Electronic supplementary material

Additional file 1: Table S1: Comparison of characteristics of included versus excluded women in the 1921-1926 cohort. (DOCX 17 KB)

Additional file 2: Table S2: Comparison of characteristics of included versus excluded women in the 1946-1951 cohort. (DOCX 17 KB)
