# Supplementary material for: Moderate agreement between self-reported stroke and hospital-recorded stroke in two cohorts of Australian women: a validation study
Source: BMC Med Res Methodol. 2015 Jan 23;15:7. doi: 10.1186/1471-2288-15-7 (PMC4320610; doi:10.1186/1471-2288-15-7)
Supplement: Supplementary file 1 — Additional file 1: Table S1: Comparison of characteristics of included versus excluded women in the 1921-1926 cohort. (DOCX 17 KB) [file 12874_2014_1158_MOESM1_ESM.docx]

**Web Table 1.** Comparison of characteristics of included versus excluded women in the 1921-1926 cohort

| Characteristic† | Included women  N = 1556 | Excluded women  N = 1000 | p-value |
| --- | --- | --- | --- |
| **Demographics** |  |  |  |
| Mean age (± SD) | 78.2 (± 1.5) | 78.3 (± 1.4) | **0.006** |
| Area of residence |  |  |  |
| Urban | 692 (45.5) | 416 (41.6) | 0.153 |
| Rural or remote | 864 (55.5) | 584 (58.4) |  |
| Marital status (N = 2553) |  |  |  |
| Married/de facto | 692 (44.5) | 428 (42.9) | 0.467 |
| Separated/divorced | 79 (5.1) | 57 (5.7) |  |
| Widowed | 729 (46.9) | 486 (48.7) |  |
| Single | 55 (3.5) | 27 (2.7) |  |
| Lives alone (N = 2547) | 743 (47.8) | 459 (46.3) | 0.456 |
| Education (N = 2498) |  |  |  |
| No formal/primary only | 389 (25.5) | 291 (30.0) | **0.002** |
| High school | 866 (56.7) | 551 (56.8) |  |
| Tertiary/trade qualification | 272 (17.8) | 129 (13.3) |  |
| Country of birth |  |  |  |
| Australia or other English speaking background | 1454 (93.4) | 902 (90.2) | **0.003** |
| Other | 102 (6.6) | 98 (9.8) |  |
| Language spoken at home |  |  |  |
| English | 1502 (96.5) | 932 (93.2) | **<0.001** |
| Other | 54 (3.5) | 68 (6.8) |  |
| **Lifestyle/risk factors** |  |  |  |
| BMI (kg/m^2^) (N = 2436) |  |  |  |
| Underweight (<18.5) | 53 (3.5) | 43 (4.8) | 0.194 |
| Acceptable (18.5-24.9) | 729 (47.4) | 413 (45.9) |  |
| Overweight (25.00-29.9) | 533 (34.7) | 296 (32.9) |  |
| Obese (≥ 30.0) | 222 (14.4) | 147 (16.4) |  |
| Physical activity‡ (N = 2516) |  |  |  |
| Nil/sedentary | 515 (49.6) | 444 (46.1) | **<0.001** |
| Low | 464 (25.1) | 265 (27.5) |  |
| Moderate | 239 (15.4) | 114 (11.8) |  |
| High | 335 (21.6) | 140 (14.5) |  |
| Smoking (N = 2295) |  |  |  |
| Never smoked | 1006 (69.7) | 551 (64.7) | **0.006** |
| Ex-smoker | 389 (27.0) | 253 (29.7) |  |
| Current smoker | 48 (3) | 48 (5.6) |  |
| **Morbidity** |  |  |  |
| History of hypertension§ (N = 2554) | 920 (59.1) | 617 (61.8) | 0.174 |
| History of heart disease§ | 624 (40.1) | 454 (45.4) | **0.008** |
| History of diabetes§ (N = 2555) | 177 (11.4) | 125 (12.5) | 0.385 |
| General health§ (N = 2553) |  |  |  |
| Excellent/very good | 579 (37.2) | 261 (26.1) | **<0.001** |
| Good | 662 (42.5) | 409 (41.0) |  |
| Fair | 294 (18.9) | 272 (27.3) |  |
| Poor | 21 (1.4) | 55 (5.5) |  |
| Assistance required for activities of daily living (N = 2540) | 121 (7.8) | 153 (15.5) | **<0.001** |

*And thus returned at least one of surveys 3, 4 or 5

†Education, country of birth and language spoken at home were collected at survey 1; smoking was collected at survey 2; self-reported history of disease included report of disease at any of surveys 1-5; all other characteristics are based on survey 3 reports (unless data was missing at survey 3, in which case information was used from either survey 4 or 5). N = 2556 unless otherwise stated.

‡Physical activity categorised in terms of minutes of moderate activity/week: Nil/sedentary = 0-10 minutes/week; low = 11-150 minutes/week; moderate = 151-300 minutes/week; high = >300 minutes/week.

§Self-reported
