# Supplementary material for: Moderate agreement between self-reported stroke and hospital-recorded stroke in two cohorts of Australian women: a validation study
Source: BMC Med Res Methodol. 2015 Jan 23;15:7. doi: 10.1186/1471-2288-15-7 (PMC4320610; doi:10.1186/1471-2288-15-7)
Supplement: Supplementary file 2 — Additional file 2: Table S2: Comparison of characteristics of included versus excluded women in the 1946-1951 cohort. (DOCX 17 KB) [file 12874_2014_1158_MOESM2_ESM.docx]

**Web Table 2.** Comparison of characteristics of included versus excluded women in the 1946-1951 cohort

| Characteristic† | Included women  (N = 2119) | Excluded women  (N = 1353) | p-value |
| --- | --- | --- | --- |
| **Demographics** |  |  |  |
| Mean age (± SD) | 52.5 (±1.5) | 52.5 (±1.5) | 0.204 |
| Area of residence (N = 3224) |  |  |  |
| Urban | 801 (38.0) | 445 (39.8) | 0.312 |
| Rural or remote | 1306 (62.0) | 672 (60.2) |  |
| Marital status (N =3465) |  |  |  |
| Married/de facto | 1736 (81.9) | 1050 (78.0) | **0.025** |
| Separated/divorced | 256 (12.1) | 200 (14.9) |  |
| Widowed | 63 (3.0) | 55 (4.1) |  |
| Single | 64 (3.0) | 41 (3.1) |  |
| Education (N = 3448) |  |  |  |
| No formal/primary only | 223 (10.6) | 236 (17.6) | **<0.001** |
| School certificate | 1027 (48.7) | 686 (51.2) |  |
| Trade/tertiary qualification | 859 (40.7) | 417 (31.1) |  |
| Country of birth |  |  |  |
| Australia or other English speaking background | 1972 (93.1) | 1184 (87.5) | **<0.001** |
| Other | 147 (6.9) | 169 (12.5) |  |
| Language spoken at home |  |  |  |
| English | 2007 (96.2) | 1223 (91.8) | **<0.001** |
| Other | 79 (3.8) | 110 (8.3) |  |
| **Lifestyle/risk factors** |  |  |  |
| BMI (kg/m^2^) (N = 3411) |  |  |  |
| Underweight (<18.5) | 24 (1.1) | 19 (1.5) | 0.416 |
| Acceptable (18.5-24.9) | 887 (41.9) | 528 (40.7) |  |
| Overweight (25.00-29.9) | 715 (33.8) | 422 (32.6) |  |
| Obese (≥ 30.0) | 489 (23.1) | 327 (25.2) |  |
| Physical activity‡ (N = 3432) |  |  |  |
| Nil/sedentary | 352 (16.7) | 298 (22.6) | **<0.001** |
| Low | 787 (37.2) | 442 (33.5) |  |
| Moderate | 450 (21.3) | 252 (19.1) |  |
| High | 525 (24.8) | 326 (24.7) |  |
| Smoking (N = 3364) |  |  |  |
| Never smoked | 1178 (55.6) | 613 (49.2) | **<0.001** |
| Ex-smoker | 686 (32.4) | 412 (33.1) |  |
| Current smoker | 255 (12.0) | 220 (17.7) |  |
| **Morbidity** |  |  |  |
| History of hypertension§ | 584 (27.6) | 392 (29.0) | 0.367 |
| History of heart disease§ | 79 (3.7) | 49 (3.6) | 0.871 |
| History of diabetes§ (N = 3471) | 53 (2.5) | 48 (3.6) | 0.073 |
| General health§ (N = 3468) |  |  |  |
| Excellent | 240 (11.3) | 119 (8.8) | **<0.001** |
| Very good | 845 (39.9) | 444 (32.9) |  |
| Good | 810 (38.2) | 551 (40.9) |  |
| Fair/poor | 224 (10.6) | 235 (17.4) |  |

*And thus returned at least one of surveys 3, 4, 5 or 6

†Education, country of birth and language spoken at home were collected at survey 1; smoking was collected at survey 2; self-reported history of disease included report of disease at any of surveys 1-5; all other characteristics are based on survey 3 reports (unless data was missing at survey 3, in which case information was used from either survey 4 or 5). N = 3472, unless otherwise state.

‡Physical activity categorised in terms of minutes of moderate activity/week: Nil/sedentary = 0-10 minutes/week; low = 11-150 minutes/week; moderate = 151-300 minutes/week; high = >300 minutes/week.

§Self-reported
